# Opposite Pathways of Cholinergic Mechanisms of Hypoxic Preconditioning in the Hippocampus: Participation of Nicotinic α7 Receptors and Their Association with the Baseline Level of Startle Prepulse Inhibition

**DOI:** 10.3390/brainsci11010012

**Published:** 2020-12-24

**Authors:** Elena I. Zakharova, Zinaida I. Storozheva, Andrey T. Proshin, Mikhail Yu. Monakov, Alexander M. Dudchenko

**Affiliations:** 1 Laboratory of General Pathology of Cardiorespiratory System, Institute of General Pathology and Pathophysiology, Baltiyskaya, 8, 125315 Moscow, Russia; monakovm@mail.ru (M.Y.M.); amdudchenko@gmail.com (A.M.D.); 2 Laboratory of Clinical Neurophysiology, Serbsky’ National Medical Research Center for Psychiatry and Narcology, Kropotkinsky per., 23, 111395 Moscow, Russia; storozheva_zi@mail.ru; 3 Laboratory of Functional Neurochemistry, P.K. Anokhin Institute of Normal Physiology, Baltiyskaya, 8, 125315 Moscow, Russia; proshin_at@mail.ru

**Keywords:** adaptation to severe hypoxia, brain structures, cholinergic forebrain projections, synaptic membrane-bound choline acetyltransferase, PNU-282987, dimethyl sulfoxide

## Abstract

(1) Background. A one-time moderate hypobaric hypoxia (HBH) has a preconditioning effect whose neuronal mechanisms are not studied well. Previously, we found a stable correlation between the HBH efficiency and acoustic startle prepulse inhibition (PPI). This makes it possible to predict the individual efficiency of HBH in animals and to study its potential adaptive mechanisms. We revealed a bi-directional action of nicotinic α7 receptor agonist PNU-282987 and its solvent dimethyl sulfoxide on HBH efficiency with the level of PPI > or < 40%. (2) The aim of the present study was to estimate cholinergic mechanisms of HBH effects in different brain regions. (3) Methods: in rats pretested for PPI, we evaluated the activity of synaptic membrane-bound and water-soluble choline acetyltransferase (ChAT) in the sub-fractions of ‘light’ and ‘heavy’ synaptosomes of the neocortex, hippocampus and caudal brainstem in the intact brain and after HBH. We tested the dose-dependent influence of PNU-282987 on the HBH efficiency. (4) Results: PPI level and ChAT activity correlated negatively in all brain structures of the intact animals, so that the values of the latter were higher in rats with PPI < 40% compared to those with PPI > 40%. After HBH, this ChAT activity difference was leveled in the neocortex and caudal brainstem, while for membrane-bound ChAT in the ‘light’ synaptosomal fraction of hippocampus, it was reversed to the opposite. In addition, a pharmacological study revealed that PNU-282987 in all used doses and its solvent displayed corresponding opposite effects on HBH efficiency in rats with different levels of PPI. (5) Conclusion: We substantiate that in rats with low and high PPI two opposite hippocampal cholinergic mechanisms are involved in hypoxic preconditioning, and both are implemented by forebrain projections via nicotinic α7 receptors. Possible causes of association between general protective adaptation, HBH, PPI, forebrain cholinergic system and hippocampus are discussed.

## 1. Introduction

One of the problems faced by practical medicine is increasing the resistance to hypoxic stress. Short episodes of moderate stress impacts of different aetiologies are capable of increasing the body’s resistance to various pathological factors. Of these, the hypoxic/ischaemic adaptive factors are of greatest interest, because it is likely that the hypoxic component forms the pathogenesis of many diseases and is the main factor in the ischaemic preconditioning effect. In this regard, a study of hypoxic (or ischaemic) preconditioning of the various organs, tissues or the organism as a whole is a high priority [1,2,3,4,5,6,7].

The brain is central to the problem of hypoxic compensatory and adaptive functions, as it is the coordinator of functions of all body organs and systems and the organ most sensitive to hypoxia. In hypoxia, the key role belongs to the autonomic respiratory and cardiovascular systems closely interrelated with the ‘respiratory center,’ groups of respiratory neurons that support respiratory rhythm [8,9,10,11,12]. They are located mainly in the medulla oblongata and pons Varolii (the caudal brainstem). The autonomic cardiorespiratory systems interact with the structures of the forebrain, and the functional significance of these connections is increasingly being explored [13,14,15,16,17]. Forebrain structures are the most unstable to ischaemic/hypoxic effects [17,18].

Research into neuronal respiratory networks in the norm and after hypoxic shock is a focus for many investigators because they are fundamental in maintaining the body’s vitality [9,10,11,12,19]. However, studies of this kind on the models of hypoxic preconditioning in the caudal brainstem are rare and seem to be non-existent for the forebrain structures. In particular, cholinergic participation is detected in the majority of the functional sites of cardiorespiratory networks (see review in [20]). However, there were no data on the participation of cholinergic system in the cardiorespiratory networks under hypoxic preconditioning.

We began to study the role of the cholinergic system of the caudal brainstem, cortex and hippocampus in hypoxic preconditioning on a one-time moderate hypobaric hypoxia model (HBH, 10–11% O_2_, 60 min). HBH has been shown to have a pronounced preconditioning effect and statistically significantly increased resistance to severe hypoxia [21,22,23]. We applied the preparative methods for the isolation of synaptic membranes and synaptoplasm from fractions of synaptosomes of the brain structures in animals who were tested under in vivo model exposures. The advantage of such methodology was repeatedly seen, since the division of synaptosomes into sub-fractions allows the study and comparison of: (1) functionally different compartments of presynapses synaptic membranes and synaptoplasm; (2) minor biochemical, membrane-bound parameters whose changes are not detected in the total fractions and at the same time they are indicators of synaptic transmitter function. Moreover, we studied separately the synaptic components of the fractions of “light” and “heavy” synaptosomes, because they include the presynapses of functionally different cholinergic neuron populations [24,25,26,27,28].

In the synaptosomal sub-fractions, the activity of membrane-bound (m) and water-soluble (s) choline acetyltransferase or acetyl-CoA: choline O-acetyl transferase (ChAT, EC 2.3.1.6) was evaluated as a cholinergic marker. Moreover, experiments in vitro convincingly show that syaptic mChAT and sChAT can also be indicators of the functional state of cholinergic synapses [29,30,31,32,33].

In our previous studies, we found that the cholinergic synaptic pool of the caudal brainstem and cortex was involved in hypoxic preconditioning. However, the functional meaning of the identified reactions for the mechanisms of adaptation was not clear in the intact (not undergone to severe hypoxia) rat group [20,26]. At the same time, we revealed a high rat-to-rat variability of HBH efficiency in resistance to severe hypoxia over a time span from 4 to 27 min [34]. Therefore, we suggested that the reaction to HBH can be based on multiple different preconditioning mechanisms. To identify and study these, physiological or neurochemical non-stressor predictors of efficiency of preconditioning were needed, but they were absent. We later found one.

In a study of sensory and motor parameters in rats with different hypoxic preconditioning efficiencies, we discovered a stable correlation between the efficiency of HBH and prepulse inhibition (PPI) in the acoustic startle reaction model, the PPI-T test [35]. PPI is a quantitative indicator of a decrease in amplitude of reaction in a situation where an acoustic signal of low intensity that does not cause a startle reaction (prepulse) precedes with a small interval (50–500 ms) before the main (intensive) stimulus [36].

PPI is the result of activity in the different brain circuits (brainstem, limbic, cortical, thalamic, strio-pallidary structures) and different synaptic mechanisms [36,37,38]. Some groups of patients with neuropsychiatric disorders display a deficiency of PPI. At the same time, PPI varies significantly in clinically normal populations. In general, acoustic startle reaction itself and PPI are thought to serve as a model of the common protective activation patterns for approaching external or internal threats (and in particular to respiration) [39]. We used the empirically found association between PPI and HBH efficiency in our studies of hypoxic preconditioning mechanisms.

Using the PPI-T test, we performed the first pharmacological experiments on the effects of nicotinic receptor (nAChRs) agonists α7 and α4β2 subtypes (respectively PNU-282987 (PNU) and RJR2403) on HBH, in low selective doses equal to their K_i_ and some higher. It is known that nAChRs are involved in hypoxic or ischaemic preconditioning in the brain and other organs, and it was most convincingly demonstrated for the α7 and α4β2nAChRs [40,41,42,43,44]. In our experiments, PNU (but not RJR2403), and more pronouncedly its solvent dimethyl sulfoxide (DMSO, 2–3%), showed a PPI-associated effect on the efficiency of HBH, the direction of which changed to the opposite at the border when PPI = 36–40% [20,45]. At the concentrations used, DMSO showed anticholinesterase activity [46] and PNU (26 and 260 nmol/kg) exerted a desensitizing effect on α7 nAChRs [47,48,49].

However, the principles for this bi-directionality of both cholinergic ligands on the efficiency of HBH were not understood. The selective potency of α7 nAChRs was also unclear. It is known that the pronounced maximum concentration and high mRNA expression of nAChRs of this subtype are in the hippocampus [47,50,51]. At the same time, in the above biochemical experiments, the cholinergic synaptic pool of the hippocampus did not show any reaction to HBH [20,26].

The aim of this exploratory investigation was to study the cholinergic mechanisms of hypoxic preconditioning in rats preliminarily tested for PPI. As before in biochemical experiments, we evaluated the effect of HBH on the activity of mChAT and sChAT in the synaptosomal sub-fractions of the synaptic membrane and synaptoplasm of the neocortex, hippocampus and caudal brainstem. We continued pharmacological experiments with PNU by testing the effects of higher doses of agonist in comparison with the effects of DMSO.

## 2. Experimental Procedures

### 2.1. Animals and Ethical Approval

Experiments were performed on male outbred albino laboratory rats aged 2–3 months (weight 200–350 g). The rats were supplied by the Light Mountains animal nursery (Russian Federation (RF)) and kept in the vivarium of the Institute of General Pathology and Pathophysiology.

All animal care and experimental procedures were carried out in accordance with the European Communities Council Directive of 24 November 1986 (86/609/EEC). All experimental protocols were approved by the Ethical Committee of the Institute of General Pathology and Pathophysiology (protocol #2 of 15.03.2019). The protocol could be provided upon request. All efforts were made to minimize animal suffering and to limit the number of animals used.

The rats were housed in a temperature-controlled room (20–24 °C) with 5–7 animals per rat cage measuring 40 × 60 cm. They had free access to food and water and were maintained with a 12 h light-dark cycle. The rats were handled for at least two consecutive days prior to starting the experimental procedures. The guillotine was used for biochemical experiments. At the end of the pharmacological experiment, the animals were euthanised via inhalation of CO_2_ using euthanasia apparatus.

### 2.2. Acoustic Startle Reaction Model

The equipment for testing PPI is described in detail [52]. The rats were tested in a soundproof room in a specialized chamber with a load cell, which recorded the amplitude of the startle reaction through a personal computer. Broadband noise with a duration of 100 ms and loudness of 110 dB was used as the main stimulus. The background-masking sound signal had a broadband noise with a loudness of 72 dB, and the prestimulus had a signal duration of 40 ms and loudness of 85 dB. The animals were placed in the chamber and were exposed to a total of 12 trials after 5 min acclimatization. The first two trials were pulse-alone trials (habituation). The remaining 10 trials were presented in pseudo-random order and included five pulse-alone trials and five pulse trials with a preceding prepulse of 100 ms in the lead-off interval. The inter-trial intervals ranged from 10 to 20 s with a mean value of 15 s. The PPI value was estimated using the formula (Am − Ap)/Am × 100%, where Am is an average reaction amplitude in the samples without the prestimulus excluding the first two (*n* = 5) and Ap is an average reaction amplitude in the samples with the prestimulus (*n* = 5). On the demonstration graphs, the PPI values are presented not in percentages, but in decimal digits.

### 2.3. Hypoxic Models

The hypoxic models were used as before [20,45]. Varying severities of hypoxia were created in the pressure chambers. The barometer of the chamber (altitude gauge) was calibrated to an altitude above sea level. The rats in the chamber were “raised” at a speed of 12–15 m/s to the adaptive altitude of 5000 m (HBH, 3.0 Pa, equivalent to 10–11% O_2_, 60 min), and for the pharmacological experiments, at a speed of 63.5–64 m/s to the critical altitude of 11,500 m (SHBH, 1.2 Pa, equivalent to 4.5% O_2_). In the critical altitude test, the resistance to hypoxia was recorded with respect to the endurance under SHBH conditions, which was the time (T) until agonal inspiration (apnoea) in combination with a loss of control of body tone.

### 2.4. Brain Tissue Preparation

The procedures of sub-synaptic fractions preparation and ChAT activity determination were performed as previously described [27].

For biochemical analysis, the rats were decapitated with a guillotine. All preparative procedures were carried out at 2–4 °C. Briefly, the brain was removed, then the caudal brainstem(medulla oblongata + pons Varolii), hippocampus and neocortex were separated and homogenized in an iso-osmotic solution containing 0.3 M sucrose, 1 mM EDTA-Na_2_ and 3 mM Tris-HCl, pH 7.4–7.5. From each structure, the light and heavy fractions of synaptosomes were isolated. Fractions of synaptosomes were obtained from the rough mitochondrial fraction by centrifugation in a discontinued sucrose gradient using a bucket rotor (84,000× *g* × 120 min, 2–4 °C) in the layers between 1.0–1.2 M sucrose densities for light synaptosomes and between 1.2–1.4 M sucrose densities for heavy synaptosomes [53]. The synaptosomes were disrupted by combined shock procedures: the synaptosome pellets were suspended in a hypo-osmotic solution containing 6 mM Tris-HCl buffer, pH 8.1 [54] (100 mg tissue/mL) and then exposed by freeze-thawing.

The synaptoplasm sub-fractions were obtained as supernatants by centrifugation from the disrupted synaptosomal fractions (14,000× *g* × 30 min, 2–4 °C). The pellets were suspended in the hypo-osmotic solution and again stratified on discontinued sucrose gradients. The synaptic membrane sub-fractions were obtained by centrifugation using the bucket rotor (130,000× *g* × 120 min, 2–4 °C) in layers between 0.6–1.2 M sucrose densities. Such purified synaptic membrane sub-fractions are free from glial, mitochondrial and synaptic vesicle contamination [55]. To achieve the iso-osmotic condition, the synaptic membrane sub-fractions were diluted 3–3.5 times with a solution containing 3 mM EDTA-Na_2_ and 9 mM Tris-HCl buffer, pH 7.4–7.5. All samples were stored at −70–80 °C until the day of the assay.

### 2.5. Choline Acetyltransferase Assay

The activity of ChAT was determined by Fonnum’s radiometric method [56]. The reactive solution was prepared on the day of the experiment. The enzymatic reaction was started by mixing sub-fraction samples with the reactive solution. The reactive mixture contained a final concentration of 0.2 mM Acetyl CoA and [1-^14^C]-Acetyl CoA with SPA 5 mCi/mmol, 300 mM NaCl, 3 mM MgCl_2_, 0.2 mM physostigmine salicylate, 10 mM choline chloride, 0.5% Triton X-100, 0.5 mg/mL albumin from bull serum, 10 mM sodium phosphate buffer, 1 mM EDTA-Na_2_, pH 7.8, and the sub-fraction samples (approximately 3.5 mg of protein) at a common volume of 0.05–0.1 mL. The reactive mixture was incubated in a water shaker at 37 °C for 30–60 min. The reaction was stopped by adding 2 mL of ice-cold stop solution (0.2 mM Acetylcholine in 10 mM sodium phosphate buffer/1 mM EDTA-Na_2_, pH 7.8) and by placing the mixture in an ice bath. Then, 1 mL of sodium tetraphenylborate solution in butyl acetate (15 mg/mL) was added and quickly subjected to intensive mixing in a shaker (500 turns/min, 4 min, room temperature). The organic phase was separated from the inorganic phase by centrifugation using the bucket rotor (1000× *g* × 15 min, 2–4 °C). The organic phase with Acetylcholine (0.5–0.7 mL) was placed into scintillation liquid for organic solutions and the radioactively synthesized [^14^C]-Acetylcholine was quantified (DPM) with a beta counter.

### 2.6. Drug Administration

PNU-282987 is a selective agonist of the α7 subtype of nAChRs. DMSO is a bipolar aprotic solvent for hydrophobic drugs. Rats given the drugs received a single intraperitoneal (IP) injection: PNU in 2% DMSO in an interval from 1.2 to 12 μmol/kg (from 323 to 3230 μg/kg) (PNU group) or 2% DMSO only (DMSO group). The control group rats received a single IP injection of saline (saline group).

### 2.7. Experimental Protocol

**Figure 1:**
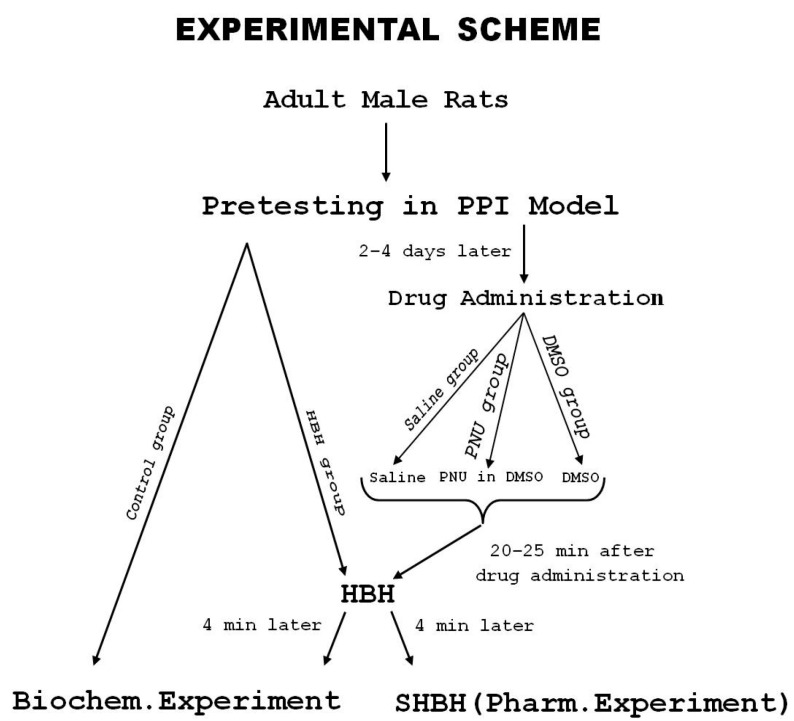
The scheme of the experiment. PPI, prepulse inhibition in acoustic startle reaction; HBH, moderate hypobaric hypoxia; SHBH, severe hypobaric hypoxia. PNU, nicotinic α7 receptor agonist PNU-282987; DMSO, solvent dimethyl sulfoxide. Biochem. Experiment, biochemical experiment; Pharm. Experiment, pharmacological experiment. For the number of animals per group, see comments in the text of this subsection (Experimental protocol).

The scheme of the experiment is presented in Figure 1. All rats (*n* = 50) were tested in the acoustic sensorimotor startle reaction model (PPI model), and the values of PPI were estimated. After that the rats were subdivided into the control (biochemical) group (*n* = 6), HBH (biochemical) group (*n* = 6), saline group (*n* = 7), PNU group (*n* = 24), and DMSO group (*n* = 7). In the biochemical experiment due to the small number of samples, rats were taken with the lowest and highest PPI values, as far as the experimental sample allowed (differences in PPI values contributed to the identification of biochemical correlates of PPI-associated HBH values). On the contrary, rats with similar low and high PPI values were selected for the control and HBH groups. Data for the saline and DMSO groups were added to those obtained in previous experiments and the total *n* in these groups was 31 and 23, respectively. In each pharmacological experimental group, rats were included for the entire range of PPI values. In the biochemical groups, they were included in equal amounts with PPI< or >40% (*n* = 3 for the control and HBH sub-groups, respectively).

Some days after PPI testing, the rats were subjected to subsequent experimental procedures. All data were obtained in a blind manner. In our experiments, each experimenter at his/her stage of obtaining experimental data did not know the key characteristics of the tested rat because an experimental group assignment was performed by a different person. Moreover, the experimental animals passed into a new environment after each test.

The rats in the HBH group were subjected to the HBH session and 4 min after its end were taken to the acute biochemical experiment. The first minutes of re-oxygenation after HBH has the most pronounced preconditioning effect [21,22,23] and we selected 4 min as optimal for our experimental conditions. The animals in the control group were taken to the biochemical experiment, bypassing the HBH and any other exposure after pretesting in the PPI model. The activity of ChAT was determined in the sub-fractions of synaptic membranes and the synaptoplasm of the neocortex, hippocampus and caudal brainstem. Accordingly, the membrane-bound mChAT activity was estimated in the synaptic membrane sub-fractions, and the water-soluble sChAT activity was estimated in the synaptoplasm sub-fractions.

The rats in the saline, PNU and DMSO groups were subjected to drug administration 20–25 min before the start of HBH training. PNU reached its maximum concentration in the brain 40–60 min after systemic administration [49]. PNU and solvents were introduced so that the peak of concentration coincided with an interval of 20–25 min from the start of HBH. The conditions for the optimal interaction of PNU and HBH were selected empirically. Four minutes after the end of the HBH training, the rats were exposed to SHBH and the values of T were estimated.

### 2.8. Reagents and Drugs

The following reagents were used: [1-^14^C]-Acetyl CoA sodium salt, Amersham Pharmacia Bioscience; Acetyl CoA sodium salt, choline chloride, naphthalene, physostigmine salicylate, sucrose, tetraphenylborate sodium salt, tris (hydroxymethyl) aminomethane sodium salt, Sigma-Aldrich; ethylene glycol, NaCl, MgCl_2_ × H_2_O and Na_2_HPO_4_ × 2H_2_O, Merck; PNU 282987, Tocris Bioscience; DMSO, LLC Tula Pharmaceutical Factory (RF); acetone, butyl acetate, dioxane, EDTA-Na_2_, PPO, POPOP, sucrose, toluol and some other reagents, REACHIM (RF).

### 2.9. Data Analysis

The results of the biochemical experiments were expressed in units of ChAT activity per minute per 1 g of raw tissue weight of the corresponding brain structure. The data obtained in the biochemical experiments (ChAT activity measure) were log-transformed and analyzed using ANOVA. The effects of PPI (low vs. high), HBH and their interaction were estimated.

The biochemical and pharmacological data were then statistically treated using the non-parametric Wilcoxon-Mann-Whitney test (u-criterion) and/or Fisher’s exact test (FET-criterion). We also performed a correlation analysis using Pearson’s *r*-criterion in Microsoft Excel and correcting formula for small selections *n* ≤ 15 [57]. An assessment of the normality of the data was carried out using STATISTICA 8.0 on significance indicators for deviation from normality according to Kolmogorov–Smirnov (parameter d and *p*-values). Deviations from normality were not detected. The differences were significant at *p* < 0.05. The Holm-Bonferroni method was used to identify significant differences when comparing the data from the saline, PNU and DMSO groups. The compared variational series in these pharmacological groups included the T values in a similar range to the PPI values (in the saline group *n* = 26 from a total *n* = 31 and in the PNU group *n* = 23 from a total *n* = 24). In the DMSO group, the one value of T calculated as an ‘outlier’ was excluded from the analysis (the ‘outlier’ exceeded the average value of variation series by 3.4 times).

## 3. Results

### 3.1. Biochemical Data Analysis

**Figure 2:**
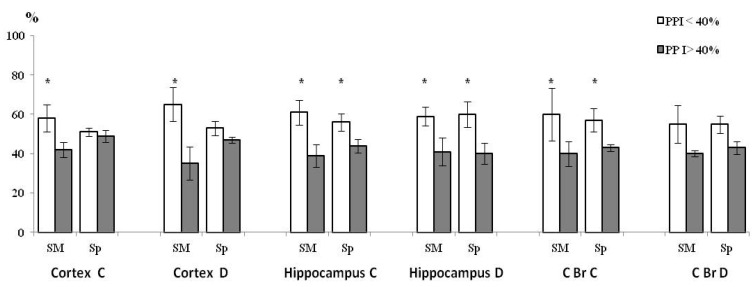
ChAT activity distribution (means ± SEM%) in the control sub-groups of rats with PPI< and >40% (*n* = 3 for each sub-group). The total activity of the enzyme in any sub-fraction of the control group is taken as 100%. Cortex, neocortex; C Br, caudal brainstem; C, the fraction of light synaptosomes; D, the fraction of heavy synaptosomes; SM, the sub-fraction of synaptic membrane; Sp, the sub-fraction of synaptoplasm. * Significant differences between values of ChAT activity in sub-groups. * *p* < 0.05, Wilcoxon-Mann-Whitney test (u-criterion) and Fisher’s exact test (FET-criterion).

**Figure 3:**
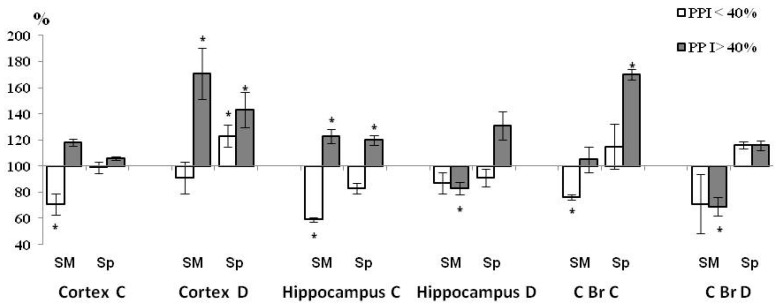
Effect of HBH on ChAT activity in synaptic sub-fractions in the HBH sub-groups in rats with PPI< and >40% 4 min after the end of hypoxic training (*n* = 3 for each sub-group). In the HBH sub-groups in each sub-fraction, ChAT activity is represented as a percentage change (means ± SEM %) relative to its activity in the corresponding control sub-group, taken as 100%. Cortex, neocortex; C Br, caudal brainstem; C, the fraction of light synaptosomes; D, the fraction of heavy synaptosomes; SM, the sub-fraction of synaptic membrane; Sp, the sub-fraction of synaptoplasm. * Significant differences between values of ChAT activity in the HBH sub-groups. * *p* < 0.05, Wilcoxon-Mann-Whitney test (u-criterion) and Fisher’s exact test (FET-criterion).

**Figure 4:**
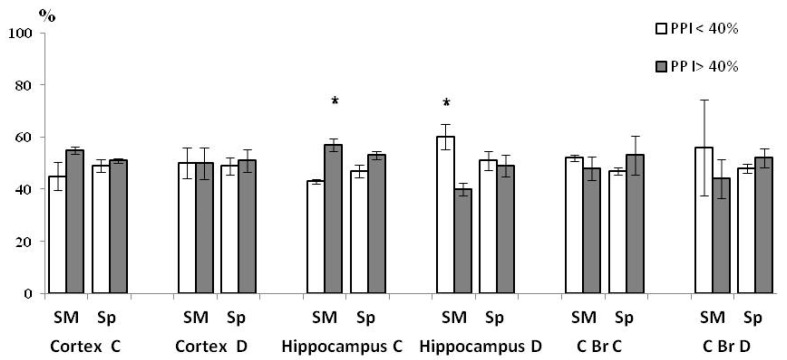
ChAT activity distribution (means ± SEM%) in the HBH sub-groups of rats with. PPI< and >40% 4 min after the end of hypoxic training. As in Figure 2, the total activity of enzyme in any sub-fraction of the control group is taken as 100%. Cortex, neocortex; C Br, caudal brainstem; C, the fraction of light synaptosomes; D, the fraction of heavy synaptosomes; SM, the sub-fraction of synaptic membrane; Sp, the sub-fraction of synaptoplasm. * Significant differences between values of ChAT activity in sub-groups. * *p* < 0.05, Wilcoxon-Mann-Whitney test (u-criterion) and Fisher’s exact test (FET-criterion).

The effects of brain structure (F(2, 12) = 4 98.29, *p* = 0.001), PPI (F(1, 6) = 10.7, *p* = 0.0177), neuron type (F(1, 6) = 4739.1, *p* = 0.001), sub-fraction (F(1, 6) = 3375.3, *p* = 0.001) and PPI × HBH interaction (F(1, 6) = 14.259, *p* = 0.00922) were presented in the report on the ANOVA tests for ChAT activity. The effects of PPI, HBH and their interaction on the ChAT activity in different brain structures, neuron types and sub-fractions are shown below (Table 1, Table 2 and Table 3 and Figure 2, Figure 3 and Figure 4).

### 3.2. The Effects of PPI, HBH and PPI × HBH Interaction on the ChAT Activity in All Data Array (ANOVA Tests)

The interaction of ChAT activity with PPI or HBH, or PPI × HBH together in all data arrays (total control and HBH rat groups) are presented in Table 1. The following significant interactions were identified: for sub-fraction type × PPI interactions in the hippocampus and caudal brainstem; for neuron and sub-fraction Type × HBH interactions in the neocortex and hippocampus; and for neuron type × PPI × HBH interactions in the hippocampus only.

### 3.3. Comparison of the Synaptic ChAT Activity and PPI Values in Total Control and HBH Groups of Rats (Pearson’s r-Criterion)

The correlations between the values of PPI and synaptic ChAT activity are presented in Table 2. In the control group, negative correlations of PPI-ChAT, mainly of PPI-mChAT, were revealed in the light synaptosomal fractions of all studied brain structures and in the heavy synaptosomal fraction of the neocortex and hippocampus.

In the HBH group, the PPI-ChAT correlational links completely disappeared in the neocortex and the caudal brainstem, but PPI-mChAT links reversed and became positive in the presynapses of the light fraction of hippocampal synaptosomes.

### 3.4. PPI-Associated Level of ChAT Activity in Synaptic Sub-Fractions in the Control and HBH Sub-Groups

In the control, in rats in the sub-group with PPI < 40%, ChAT activity was higher than in rats in the sub-group with PPI > 40% in all 12 studied sub-fractions. In the eight sub-fractions, this separation was complete and significant (Figure 2). It should be noted that in the neocortex in the sub-fractions of both synaptosome fractions, the complete intergroup separation was manifested only in the mChAT activity. Despite the fact that the ratio between the activity of mChAT and sChAT varied greatly in the different brain structures and types of synaptosomal fractions, an overwhelming proportion of activity belonged to sChAT in all studied fractions (Table 3). Therefore, the differences revealed in the neocortex would go unnoticed when the enzyme was studied at the level of the initial synaptosomal fractions.

Under the influence of HBH, compared with the control sub-group (Figure 3) in rats with PPI < 40% in all three experiments of the HBH sub-group, mChAT activity decreased in the light fractions of synaptosomes in the neocortex, hippocampus and caudal brainstem, and sChAT was activated in the heavy fraction in the neocortex. In rats with a PPI > 40% in all three experiments of the HBH sub-group, ChAT activity increased in both sub-fractions of the neocortical heavy synaptosomes and hippocampal light synaptosomes, as well as in the sub-fraction of synaptoplasm (sChAT) of light synaptosome in the caudal brainstem. In the sub-fractions of synaptic membranes of heavy fractions in the hippocampus and caudal brainstem, enzyme activity (mChAT) decreased.

As a result of HBH-initiated changes in ChAT activity (Figure 4), differences between sub-groups in this indicator disappeared in the sub-fractions of neocortex and caudal brainstem. In the hippocampus, differences between sub-groups in the mChAT activity reversed in the light synaptosomal fraction and remained as in the control group in the heavy fraction of synaptosome.

### 3.5. Pharmacological Data Analysis

In the saline group, the T values obtained in this study (*n* = 7) supplemented the array of previously obtained data on the PPI-T test [34,45], and still more strengthened the level of its probability (r = −0.466, *p* < 0.01, *n* = 31). The T values in the DMSO group of rats (2% DMSO) were combined and supplemented previously obtained data on 2% or 3% DMSO [34,45]. This confirmed the positive correlation of PPI-T in this group (r = +0.424, *p* < 0.05, *n* = 22). PNU was tested for the first time in doses of 1.2 (*n* = 9), 2 (*n* = 8), 4 (*n* = 4) and 12 μmol/kg (*n* = 3) and presented similar results in this dose range, so the data were combined and showed a negative correlation of PPI-T in the PNU group (r = −0.693, *p* < 0.001, *n* = 24).

### 3.6. PPI-Associated Effects of PNU and DMSO on HBH Efficiency

**Figure 5:**
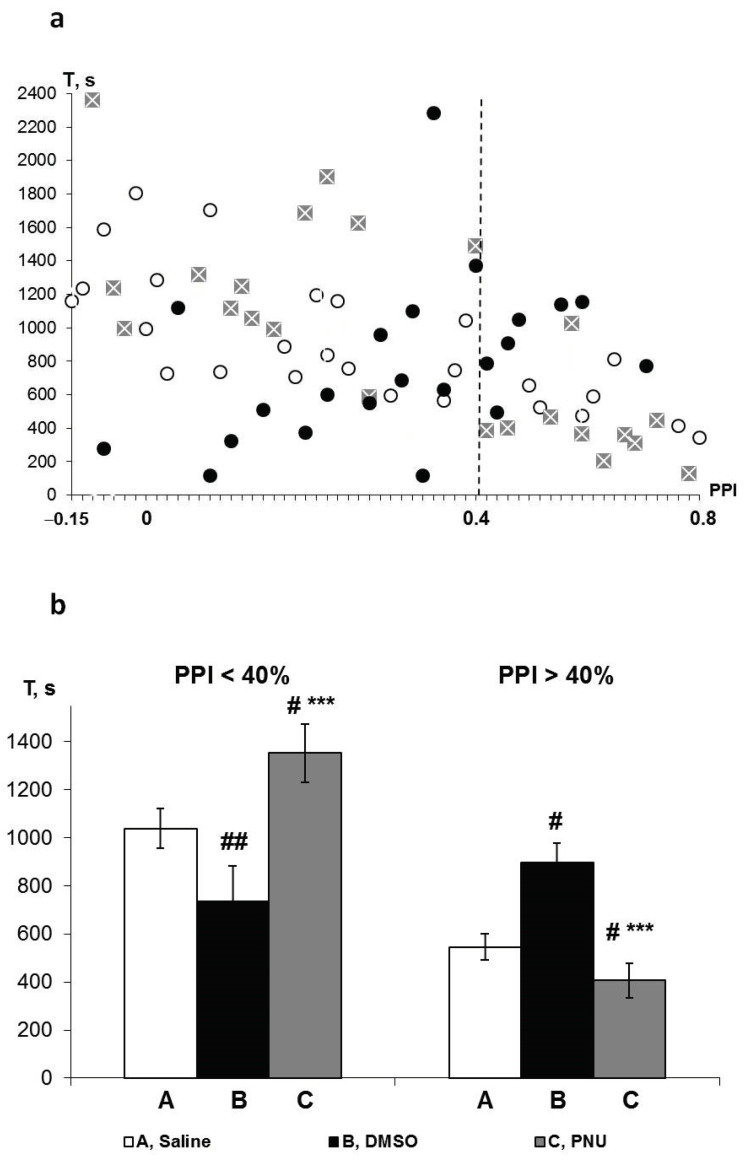
PPI-associated effects of the IP administration of α7 nAChR agonist PNU and its solvent DMSO on the HBH efficiency of hypoxic preconditioning. T, the time before apnoea (SHBH resistance criterion). (**a**) individual values of T corresponding to own PPI. White round markers, the T values in saline group (HBH only, *n* = 26); crosses in grey squares markers, the T values in PNU group (PNU in DMSO + HBH, *n* = 23); black round markers, the T values in DMSO group (DMSO + HBH, *n* = 22). Vertical dotted line indicates the value of PPI = 0.40, an approximate border of the reversal of direction of action of both cholinergic ligands on HBH efficiency. For Pearson’s *r*-criterion values see “Results”, subsection “Pharmacological data analysis”. On the abscissa axis, the PPI values are distributed in proportion to the density of T values. (**b**) the average T values (means ± SEM) in the sub-groups of rats with PPI < 40% and PPI > 40%; A, saline sub-groups; B, DMSO sub-groups; C, PNU sub-groups. # significant differences with the saline sub-group; * significant differences with the DMSO sub-group. # *p* < 0.05; ## *p* < 0.025; *** *p* < 0.01. The Wilcoxon-Mann-Whitney test (u-criterion) and Fisher’s exact test (FET-criterion). *p* values are presented as amended by the Holm-Bonferroni method. For saline, PNU, DMSO sub-groups with PPI < 0.4 or < 40% *n* = 19, 13, 15 and with PPI > 0.4 or > 40% *n* = 7, 10, 7, respectively.

The individual T values of all three groups of rats are displayed in the combined Figure 5a. This shows that a change in the direction of influence of both PNU and its solvent DMSO on the T values in the saline group occurred at the border PPI of approximately 40%. PNU predominantly potentiated the effects of HBH in rats with lower PPI values and reduced the effects of HBH in rats with higher PPI values. DMSO, by contrast, predominantly reduced the effects of HBH in rats with lower PPI values and potentiated them in rats with higher PPI values.

Therefore, we subdivided the pharmacological study data into sub-groups with PPI < 40% and PPI > 40% and compared the quantitative differences in average T values between the saline, PNU and DMSO sub-groups (Figure 5b). According to the averaged data, the influence of both PNU and DMSO on the T values in both saline sub-groups was significant and opposite with respect to each other. Thus, the greatest differences were observed between the PNU and DMSO sub-groups.

## 4. Discussion

An analysis of the data obtained in the biochemical experiments revealed both the association between the activity of ChAT and the level of PPI in the intact animals, as well as the multi-directional effect of HBH on the activity of this enzyme in rats with high and low PPI (interaction of HBH × PPI detected by ANOVA).

In the control rat sub-groups, it was found that the activity of synaptic ChAT in the sub-group with PPI < 40% was higher than in the sub-group with PPI > 40% in all three brain structures in the majority of sub-fractions. The legitimacy of the revealed differences was supported by a high level of negative correlation between PPI values and ChAT activity in the corresponding sub-fractions in the total control group.

Our results concerning the involvement of cholinergic systems in the PPI mechanisms are consistent with studies performed on the neocortex, hippocampus and brainstem [58,59,60,61]. In total, these and other studies reported the ambiguous relationships between PPI and cholinergic activity, as well as the difference in the mechanisms of these relationships in the brain of intact animals and under conditions of experimental impact [62,63,64].

The effect of HBH combined with HBH × PPI interaction was observed in the light fraction of synaptosomes in the hippocampus. This synaptosomal fraction was the only one in which significant PPI-mChAT and PPI-sChAT correlations were observed in HBH-treated rats. However, the directions of these correlations after HBH changed to opposite relative to those observed in intact animals. In the HBH-treated rats in this light fraction of the hippocampus, the activity of mChAT decreased in the sub-group with PPI < 40% and increased in the sub-group with PPI > 40%. This led to a significant inversion in the ratio of the activity of mChAT between sub-groups and compared with the control sub-groups. In the hippocampus, in the heavy fraction of synaptosomes, differences in mChAT activity persisted and deepened as a result of an even greater decrease in enzyme activity in the sub-group with PPI > 40%. In contrast, due to changes in mChAT and/or sChAT activity in other sub-fractions (all sub-fractions of the neocortex and caudal brainstem and the sub-fractions of synaptoplasm in the hippocampus), the ChAT activity after HBH was equalized between the sub-groups.

It has been shown in in vitro studies that in the nerve endings, the mChAT activity is associated with the quantum secretion of acetylcholine [29,33,65,66]. The activity of mChAT (but not sChAT) depends on factors regulating quantum secretion such as α-Ca^2+^/calmodulin-dependent protein kinase II, vesicular acetylcholine transporter, ion balance in the vesicular Ca^2+^/H^+^ antiport (proton gradient), ions K^+^, Zn^2+^, Cl^−^ [30,31,33,66,67,68]. These studies show a high probability that the activation of mChAT indicates the activation of a cholinergic secretory function, and the inhibition of mChAT indicates its inhibition in the corresponding presynaptic populations. Changes in the ChAT activity may also reflect corresponding changes in the number of cholinergic nerve endings (for example, in our experimental conditions, a reduction of presynapses is possible, which can occur in minutes). However, in any case, the activity of ChAT, especially mChAT, is an indicator of the direction of changes in cholinergic influences of the corresponding presynaptic population.

Thus, in the present study, HBH-initiated multidirectional changes in the activity of mChAT were observed selectively in the hippocampus and only in the light fraction of synaptosomes. These changes reflect the inhibition of the quantum secretion of acetylcholine (decrease in cholinergic influence) in the sub-group with PPI < 40% and its activation in the sub-group with PPI > 40%. This PPI-associated multidirectional shift in cholinergic secretory function is consistent with the oppositely directed effects of cholinergic ligands on the efficiency of HBH.

DMSO inhibited the preconditioning effect of HBH in rats with PPI < 40% and potentiated it in rats with PPI > 40%. Analysis of the data concerning the penetration of DMSO through blood–brain barrier and its effects at different concentrations [46,69,70,71,72] leads to the conclusion that in our experiments only the anticholinesterase action of this solvent took place in used low doses [45].

The effects of PNU were significantly different to those of DMSO in both sub-groups of rats. We obtained similar, but less pronounced results with even lower doses of PNU in a previous study (26 and 260 nmol/kg, IP) [45]. In that article, based on the data found in the literature, we concluded that PNU desensitized α7 nAChRs. The effects of PNU observed in the present study may also be due to the desensitization of α7 nAChRs. The desensitizing action of PNU in similar doses was also assumed in another study [73]. The effects of PNU observed in the present study overpowered the anticholinesterase effects of DMSO. This showed a dose-dependent desensitizing action of PNU. At the lower doses in the previous study, PNU also acted opposite to the action of DMSO, but the efficiency of the action was small. In both PNU sub-groups, the mean T values occupied an intermediate position between those in the HBH and DMSO sub-groups [45]. Note that the desensitization is regarded as a natural pathway of modulation of consolidated long-term potentiation (LTP), one of the forms of neural plasticity in the hippocampus [74].

The concentration of α7 nAChRs in the hippocampus exceeds that of any other brain structures [47,50,51]. This suggests that in our pharmacological experiments, hippocampus may be one of the main targets of PNU and, accordingly, supports the correspondence of our biochemical and pharmacological data. In contrast, the selective agonist of α4β2 sub-type of nAChR RJR2403 at doses equivalent to doses of PNU had no effect on the efficiency of HBH [20].

The fact that HBH-initiated inter-group differences in mChAT activity were selectively revealed in the hippocampus, and this was observed precisely in the light fraction of synaptosomes, should be discussed taking into account the participation of this brain structure in system mechanisms of plasticity and memory [75,76,77,78,79]. The hypoxic preconditioning effect is a short-term adaptation to severe hypoxic impacts. The adaptive effect of HBH persists throughout the day [21,22,23]. In time, the preconditioning corresponds to the so-called “intermediate” memory, which is the “bridge” between working and long-term memory in learning [80]. Again, as in the learning methodology, repeated adaptive hypoxic training leads to the formation of a prolonged protective track [81,82,83,84,85,86], i.e., to the consolidation of long-term memory. Thus, the preconditioning trail is a kind of short-term memory, which provides an involuntary protective reaction of the body against a damaging stressful effect.

Accumulated experimental data show that the hippocampus is responsible for filtering and maintaining new and significant signals of any modality. In particular, the hippocampus was shown to participate in the adaptive plasticity of visceral functions in the course of defensive memory formation [87].

It has been substantiated that in the hippocampus, theta rhythm can play a filtering role, providing protection against interfering signals arriving during the processing of significant stimuli [75,88,89,90]. Theta rhythm in the hippocampus is generated by cholinergic and GABAergic pacemaker projection neurons from the nuclei of the medial septum and Broca’s diagonal band [75,88,89,91] and most of those cholinergic neurons have pacemaker activity [75,88]. Both structures are the main source of cholinergic influences in the hippocampus and are actively involved in memory processes [75,88,92,93,94]. In other words, the cholinergic projections onto the hippocampus are involved in providing synchronized tonic influences necessary for its neural network to process a new significant signal into a short-term memory trace.

In the neocortex and hippocampus, there are two main sources of cholinergic influences, the subcortical nuclei of the forebrain and interneurons. According to the literature, these sources vary greatly in size. The power of influence of cholinergic projective neurons is 5 (in the neocortex)–30 times (in the hippocampus) higher than that of interneurons. In the light and heavy synaptosomes of both the neocortex and the hippocampus, the activity of ChAT, a marker of cholinergic neurons, has a similar proportion. Together with usually detected differences in the response of ChAT in these synaptosomal fractions to the experimental impacts, this suggests the concentration of presynapses of cholinergic projective neurons in the light fractions of synaptosomes and presynapses of interneurons—in the heavy fraction of synaptosomes [24,25,28]. Hence, it is highly likely that the reciprocal changes in the activity of mChAT, observed after HBH in the hippocampus, took place in the presynapses of cholinergic projective neurons.

Key participation of the cholinergic projections of the hippocampus in the mechanisms of hypoxic preconditioning and the dependence of the direction of their functional response to HBH on the PPI values of an intact animal is the main finding of our study.

In electrophysiological and optogenetic studies it has been shown that the cholinergic component of theta rhythm in the hippocampus is responsible for its power (amplitude) and suppression of competing for non-theta mechanisms [75,89,90,95]. In our study, cholinergic activation was observed in the sub-group of rats with PPI > 40%. In the sub-group of rats with PPI < 40% (those with increased intact cholinergic activity), HBH initiated inhibition. Since the preconditioning effect of HBH was observed in both sub-groups, it could be proposed that the mechanisms of adaptive plasticity initiated by mild hypoxia in the hippocampus include stabilization of the activity of cholinergic projections near the several optimal levels that may be necessary for synchronization a theta rhythm generation.

It should be noted here that the differences between our sub-groups in terms of cholinergic activity can be not only quantitative, but also qualitative, i.e., they may differ in the pattern of their inputs. This is an under-researched topic. According to the literature, the effects of cholinergic stimulation or inhibition depend on the composition of the connections of cholinergic projections with the hippocampal GABAergic interneurons of the first and second-order and with the pyramidal (glutamate) neurons [93,96,97], as well as with the fast and non-fast (slow) spiking GABAergic interneurons [98,99]. In particular, cholinergic modulation in the neural networks depends on the location of the nicotinic activity, primarily α7 nAChRs [99,100,101], and the low and high cholinergic states also determine the specificity and power of theta and LTP generation [98,99].

When sensory information arrives, the first perceiving structure is the prefrontal neocortex and the hippocampal-neocortical connectivity drives sensory processing [75,98,102]. The hippocampus becomes involved in the processing of information at the later stages, providing the prolonged action of new and significant signals [75,102]. Obviously, the neuronal activity of brainstem structures, reflecting the registration and transmission of sensory information [9,10,11,12,19], preceded both the neocortical and hippocampal phases of its perception and processing. Apparently, in our study, we recorded the phase of maintaining the signal in the hippocampus. Therefore, the inter-group alignment of cholinergic synaptic activity in the neocortex and caudal brainstem may reflect inhibition of the previous functional activity of these structures.

In the hippocampus, in addition to reciprocal changes in the HBH sub-groups in the light fraction of synaptosomes, there were changes in the activity of mChAT in the heavy fraction of synaptosomes in the sub-group with PPI > 40%. The direction of changes was opposite to that of the activity of mChAT in the light fraction in this sub-group of rats. However, it amounted to only 4% of the changes in the light fraction. The activity of mChAT (nmol ACh/1 min/1 g tissue) in the heavy and light fractions was, respectively, 0.0448 ± 0.0016 versus 0.787 ± 0.032 in the control sub-group and 0.0374 ± 0.0022 versus 0.966 ± 0.041 in the HBH sub-group. The functional properties of cholinergic presynapses of the hippocampal heavy fraction (presumably belonging to interneurons) have not been studied practically. According to our data, the cholinergic presynapses from heavy fractions of the neocortex and hippocampus are actively involved in cognitive functions [24,27,103] and the hippocampal presynapses can be involved in the storage of long-term spatial memory [103]. The involvement of hippocampal cholinergic interneurons in the mechanisms of memory is confirmed in a study using optogenetic engineering [104]. In the present study, the heavy synaptosomal fraction was the only one in which the cholinergic presynapses retained native differences between sub-groups and even deepened them. Obviously, these presynapses carry some specific functional load in conditions of hypoxic preconditioning. However, this issue needs further research. Nevertheless, the inhibition of the functional activity of neurons involved in the consolidation of long-term memory can be a means of protection against extraneous stimuli in the sub-group with PPI > 40%.

The association that we have discovered between PPI and HBH efficiency is currently purely empirical. However, the comparison of our results with some literature data indicates the existence of its neurological substrate, at least in relation to cholinergic projection neurons of the medial septum. In our study, different statistical methods revealed a significant interaction of PPI-ChAT and HBH effects on ChAT activity in the control and/or HBH group in the presynapses of light fraction synaptosomes of the hippocampus (in which, we recall, the projective cholinergic neurons are concentrated from the medial septum and Broca’s diagonal band). The medial septum participates in PPI mechanisms [105]. Recently, the septal projection cholinergic neurons have been shown to be a key cell type involved in PPI [106]. In turn, the pedunculopontine tegmantal area is one of the key structures for PPI [36] and was shown to be involved in the cardiorespiratory adjustments under increased metabolic demands and hypoxia [107].

The anatomical basis of such properties of this area is mainly the relay neurons of the laterodorsal (LDT) and pedunculopontine (PPT) tegmental nuclei. LDT and more intensively PPT send the plurality of the fibers to both the pontine and medulla oblongata nuclei and also to the higher brain structures, including the basal forebrain nuclei [108,109,110]. The neurons of PPT and LDT are the main switch between the cortical cholinergic projective neurons and brainstem formations [111]. Some neurons in these tegmental nuclei send the projections to both the basal ganglia and medulla oblongata [110].

The inherited PPI level in rats was previously shown to be a predictor of response to such adaptive impact as neonatal handling on the working memory and the volume of the hippocampus [112]. PPT belongs to the brainstem system which synchronizes hippocampal theta activity [95,113]. However, the question of whether the identified association reflects common systemic adaptive mechanisms in health and disease needs further research.

## 5. Conclusions

Two oppositely directed PPI-associated cholinergic mechanisms of hypoxic preconditioning in a single HBH session were identified. This phenomenon substantiates by the results of both biochemical and pharmacological experiments.

We have proved that in the preconditioning mechanisms of HBH, the hippocampal synaptic ChAT activity of the projective neurons from the forebrain nuclei reversed the interaction with PPI inherent in the intact brain, as well as cholinergic ligands reversed the direction of their action on HBH efficiency via hippocampal α7 nAChRs at the PPI border of approximately 40%.

In contrast to events in the hippocampus, the PPI-dependent inter-group differences in the synaptic ChAT activity inherent in the intact brain were leveled in the neocortex and caudal brainstem in the HBH sub-groups.

Thus, according to our data, the pronounced differences between rats with high and low PPI in the HBH-induced changes of cholinergic activity were observed only in the hippocampus.

The associations we found between the efficiency of HBH, the level of PPI and remodeling of the cholinergic systems of the hippocampus, as well as of the neocortex and caudal brainstem, may reflect the functional patterns of multiple adaptive-compensatory mechanisms, in which the hippocampus acts as a physiological substrate of cross-correlation relations between the preconditioning impulses to stressful effects of different modalities.

The bi-directional effects of the α7 nAChRs agonist PNU and its solvent DMSO on the resistance of the body to oxygen deficiency in subjects with high and low PPI values revealed in this study raise the possibility of realizing the principles of personalized medicine, taking into account the PPI-associated effects of drugs on patients.

## Figures and Tables

**Table 1:** The effects of PPI, HBH and PPI × HBH interaction on ChAT activity in different brain structures, neuron types and sub-fractions (ANOVA tests).

Brain Structure	Sub-Fraction	Total Control and HBH Groups		
		PPI	HBH	PPI × HBH
Neocortex	C, SM	F(1, 4) = 0.302, *p* = 0.60	F(1, 4) = 0.95, *p* = 0.42	F(1, 4) = 5.8, *p* = 0.050
	C, Sp	F(1, 4) = 0.004, *p* = 0.96	F(1, 4) = 0.058, *p* = 0.83	F(1, 4) = 0.93, *p* = 0.44
	D, SM	F(1, 4) = 3.9, *p* = 0.12	F(1, 4) = 3,9, *p* = 0.11	F(1, 4) = 2.9, *p* = 0.14
	D, Sp	F(1, 4) = 0.19, *p* = 0.69	***F(1, 4) = 12.6 p = 0.013***	F(1, 4) = 0.72, *p* = 0.43
Hippocampus	C, SM	***F(1, 4) = 4.21, p = 0.075***	***F(1, 4) = 10.81, p = 0.010***	***F(1, 4) = 63.1, p = 0.0002***
	C, Sp	***F(1, 4) = 51.9, p = 0.0004***	***F(1, 4) = 45.88, p = 0.0005***	***F(1, 4) = 88.5, p = 0.00008***
	D, SM	***F(1, 4) = 10.36, p = 0.019***	F(1, 4) = 1.15, *p* = 0.33	F(1, 4) = 1.10, *p* = 0.34
	D, Sp	*F(1, 4) = 4.32, p = 0.079*	F(1, 4) = 0.81, *p* = 0.39	F(1, 4) = 297, *p* = 0.13
Caudal Brainstem	C, SM	***F(1, 4) = 9.3, p = 0.023***	F(1, 4) = 1.07, *p* = 0.34	F(1, 4) = 3.19, *p* = 0.12
	C, Sp	F(1, 4) = 0.17, *p* = 0.82	F(1, 4) = 2.8, *p* = 0.14	F(1, 4) = 1.8, *p* = 0.22
	D, SM	F(1, 4) = 0.22, *p* = 0.66	F(1, 4) = 2.4, *p* = 0.19	F(1, 4) = 0.08, *p* = 0.91
	D, Sp	F(1, 4) = 1.09, *p* = 0.32	F(1, 4) = 0.11, *p* = 0.83	F(1, 4) = 3.15, *p* = 0.12

C, the fraction of light synaptosomes; D, the fraction of heavy synaptosomes; SM, the sub-fraction of synaptic membrane; Sp, the sub-fraction of synaptoplasm. Bold and italics represent the significant interactions of the ChAT activity with PPI and/or HBH. In the total control and HBH groups, *n* = 12 for each sub-fraction.

**Table 2:** Correlation between ChAT activity and PPI values in the total control and HBH groups of rats, Pearson’s *r*-criterion.

Brain Structure		Neocortex				Hippocampus				Caudal Brainstem			
Sub-Fraction		C, SM	C, Sp	D, SM	D, Sp	C, SM	C, Sp	D, SM	D, Sp	C, SM	C, Sp	D, SM	D, Sp
Control Group *n* = 6	*r*	***−0.849***	−0.305	***−0.959***	−0.279	***−0.964***	***−0.821***	***−0.914***	***−0.981***	***−0.951***	***−0.844***	−0.496	−0.753
	*p*	***<0.05***	>0.05	***<0.01***	>0.05	***<0.01***	***<0.05***	***<0.02***	***<0.001***	***<0.01***	***<0.05***	>0.05	>0.05
HBH Group *n* = 6	*r*	+0.219	−0.096	−0.134	−0.190	***+0.920***	***+0.892***	***−0.871***	−0.349	−0.455	+0.246	−0.177	+0.259
	*p*	>0.05	>0.05	>0.05	>0.05	***<0.01***	***<0.02***	***<0.05***	>0.05	>0.05	>0.05	>0.05	>0.05

C, the fraction of light synaptosomes; D, the fraction of heavy synaptosomes; SM, the sub-fraction of synaptic membrane; Sp, the sub-fraction of synaptoplasm. Bold and italics represent the significant correlation between ChAT activity and PPI values.

**Table 3:** ChAT activity values in the control sub-groups of rats with PPI < and > 40% and the interaction of PPI with neuron type in the total control group in different brain structures and sub-fractions (ANOVA tests).

Brain Structure		Cortex				Hippocampus				Caudal Brainstem			
Sub-Fraction		C, SM	C, Sp	D, SM	D, Sp	C, SM	C, Sp	D, SM	D, Sp	C, SM	C, Sp	D, SM	D, Sp
PPI < 40%													
Control sub-group *n* = 3	M	3.850	13.480	0.0382	0.973	1.241	12.025	0.0611	0.787	0.273	1.868	0.0287	0.730
	± SEM	0.251	0.637	0.004	0.059	0.016	0.595	0.008	0.021	0.014	0.081	0.004	0.052
PPI > 40%													
Control sub-group *n* = 3	M	2.960	13.00.	0.0189	0.860	0.793	8.103	0.0451	0.580	0.182	1.391	0.0209	0.583
	± SEM	0.205	0.613	0.0003	0.058	0.031	0.081	0.002	0.029	0.021	0.071	0.001	0.010
PPIxneuron type													
Total Control group *n* = 6	PPI	F(1,4) = 0.28, *p* = 0.24	F(1,4) = 0.13, *p* = 0.82	***F(1,4) = 193, p = 0.005***	F(1,4) = 0.7, *p* = 0.51	***F(1,4) = 33.2, p = 0.028***	***F(1,4) = 95.2, p = 0.01***	F(1,4) = 2.56, *p* = 0.27	F(1,4) = 14, *p* = 0.1	F(1,4) = 13.8, *p* = 0.1	F(1,4) = 11.8, *p* = 0.07	F(1,4) = 0.22, *p* = 0.69	F(1,4) = 2.4, *p* = 0.26

Data represent means ± SEM of the ChAT activity in nmol of synthesized ACh per 1 min per 1 g tissue. M, mean value. The line below, for the ChAT activity in total control rat group, the analysis revealed significant neuron type × PPI interactions in the neocortex and hippocampus. C, the fraction of light synaptosomes; D, the fraction of heavy synaptosomes; SM, the sub-fraction of synaptic membrane; Sp, the sub-fraction of synaptoplasm. Bold and italics represent the significant interactions of the ChAT activity with PPI.

## Data Availability

The data presented in this study are available on request from the corresponding author.
